# Effect of a Marking Pheromone and Population Density on Ladybird Larval Development and Adult Body Mass

**DOI:** 10.3390/insects17030317

**Published:** 2026-03-16

**Authors:** Lucas Fernandez, Oldřich Nedvěd

**Affiliations:** 1 Faculty of Science, University of South Bohemia, 37005 České Budějovice, Czech Republic; fernal01@prf.jcu.cz; 2 Biology Centre of Czech Academy of Sciences, Institute of Entomology, 37005 České Budějovice, Czech Republic

**Keywords:** semiochemicals, Coccinellidae, alkanes, hydrocarbons, population density

## Abstract

Females of predaceous ladybirds use sensing chemicals in larval tracks as an oviposition-deterring pheromone to avoid cannibalism of eggs. We hypothesized that larvae would also respond to the presence of conspecific tracks by slowing their developmental rate and delaying pupation, thereby reducing the time spent as a defenseless pupa in the presence of feeding conspecifics. We reared larvae of the harlequin ladybird *Harmonia axyridis* in dishes that were replaced daily by a clean one (C) or continuously in a dish with larval tracks accumulated (P). We used three larval densities (1, 4, 8 larvae per dish) for both regimes (C1, C4, C8, P1, P4, P8). We measured the developmental time of the fourth larval instar, pupae, and fresh adult body mass. Developmental time increased at the highest density in the combination C8 but remained unchanged across densities in the dishes with pheromone (P1–P8). Body mass was significantly lower at the highest density in both regimes (C8, P8) and was slightly higher in the presence of pheromone (P). Ladybird larvae respond independently of their density and of the presence of pheromones. The compounds present in the tracks, previously known as oviposition-deterring pheromone, may be associated with a buffering effect on density-related developmental delays.

## Simple Summary

Larvae of ladybird beetles living in a high number together can compete for food and cannibalize other larvae. They can feel their density as a number of encounters and smell their footprints. We reared larvae of Harlequin ladybird *Harmonia axyridis* in three densities (1, 4, 8 in a dish) and either in dishes cleaned daily or with accumulated smell. We measured the time of development of larvae, and the body mass of the adults subsequently emerged. Larvae in dirty dishes kept developing fast even at high density, while larvae in clean dishes developed slowly at the high density. They thus avoided becoming defenseless pupae in the presence of other feeding larvae. Adult beetles that resulted from larvae living at high density were much lighter than at low density. This effect was probably caused by the frequency of meeting other larvae in a dish, not by the amount of the chemicals in footprints, because beetles from dirty dishes were slightly heavier. High-density populations may begin to self-regulate due to negative effects on growth and survival, reducing the risk of overpopulation.

## 1. Introduction

The beetle family Coccinellidae (Coleoptera) is recognized for its striking colors but is also known worldwide for its significant impact on ecosystems and its role in pest control, sparking great interest both scientifically and in agro-industrial applications [1]. Coccinellids are natural predators, especially of aphids and scale insects, although some species are phytophagous or mycophagous [2]. Many species exhibit complex trophic interactions, including intraguild predation (IGP) [3] and cannibalism [4].

Reproduction begins once the adult female lays her eggs, in the tribe Coccinellini usually in clusters near a colony of aphids or other food source [5]. The eggs are bright yellow or orange and hatch within three to seven days, primarily depending on the temperature to which they are exposed [6]. The larvae pass through four instars prior to pupation.

Both eggs and pupae are immobile; they do not actively defend themselves, and they are thus more prone to be cannibalized than larvae [7]. Older ladybird larvae may also attack and feed on smaller larval instars [8]. Adults and larvae of *Coleomegilla maculata* and *Propylea quatuordecimpunctata* attacked eggs, first and second instars of the invasive harlequin ladybird, *Harmonia axyridis* [9]. Although selection for faster growth and development should minimize the risk of being cannibalized as an egg or a small larva, it can increase the risk of being cannibalized as a pupa by slower conspecific fourth instar larvae. Besides cannibalism, simple competition for food can be a driver for faster development. But this competition will be strong only when food is scarce, and thus the fast development would lead to smaller adults.

One form of intraspecific communication detected in insects provides protection of eggs and young larvae against cannibalism and IGP by older larvae or simply against competition for a limited food source. It is known as the oviposition-deterring pheromone (ODP) or allomone. ODP in fruit flies consists of chemicals released by adult individuals that affect female decisions regarding egg-laying, leading other females to avoid depositing eggs in the same fruit [10].

Similar effects have been detected in aphidophagous predators *Coccinella septempunctata* and *Chrysopa oculata* [11]. In Coccinellidae, ODP are deposited by larvae. Females do not lay eggs or lay less eggs in the presence of either conspecific or heterospecific larval tracks. This effect persists from one day to one month [12]. Chemical analyses of the compounds responsible for this communication included short-chain hydrocarbons and esters [13]. Initial studies on this phenomenon in *H. axyridis* also demonstrated that chemical markers could deter females from laying eggs, thus reducing resource competition and cannibalism among larvae [14].

Population dynamics of systems with ladybirds and aphids can be better specified as a metapopulation model of aphid–ladybird interactions than by classical Lotka–Volterra models of the predator–prey relationship [15]. Selection should favor mechanisms that enable predators to avoid reproducing in patches with insufficient prey and those already occupied by predators [16]. Aphids have an extremely short life cycle resulting in a high generation time ratio between ladybirds and aphids, compared, e.g., to the ratio between ladybirds and coccids [17]. Optimization of the oviposition sites in plants with aphids then favor using compounds deposited by conspecifics as a clue to search for alternative food patches [18].

As mentioned above, pupae are also at risk of IGP and cannibalism. We expect that ODP may also influence the larval developmental rate and resulting body size of pupae and adults. Larvae could either accelerate their development to avoid competition for food or decelerate it to not become defenseless pupae in the presence of voracious larvae. Such type of stress represented by an exposure of either larvae or adults of *Cheilomenes sexmaculatus* to IGP risk by *H. axyridis* decreased either adult or offspring body size [19].

Population density of moderately mobile ladybird larvae should be important in both competition for food and in cannibalism or IGP. Cannibalism accordingly increased with larval density in *Cycloneda sanguinea* and *H. axyridis* [20]. However, the study suggested that not all attacks on conspecifics were driven by hunger.

The aim of this research was to investigate whether exposure to larval tracks and the intensity of physical larval presence due to population density would affect the development of *H. axyridis* larvae and the body mass of resulting adults. The development times of the last larval instar and the pupal stage and adult body mass were measured in the presence of diverse amounts of larval tracks pheromone and at various population densities.

## 2. Materials and Methods

We maintain a laboratory stock of the ladybird *H. axyridis* named Stáňa, which belongs to the color form *novemdecimsignata* (otherwise known as *succinea*) at 25 °C, 16:8 LD [21]. We fed them with frozen *Ephestia* eggs and aphids *Acyrthosiphon pisum* reared on *Vicia faba* and provided them with water in cotton pads and corrugated paper for oviposition. Eggs were separated from the adults, and larvae were reared in groups of five in 10 cm diameter Petri dishes. Water and food were renewed once a day until the larvae reached the third instar. Then we checked them twice a day (once every 12 h) until larvae reached the fourth instar. Then the larvae of the same developmental stage (within a 12 h interval) were separated into new clean Petri dishes with sufficient water and food.

We assigned the larvae to six combinations of the levels of two factors. One factor was a dish regime with two levels: Clean (C, dish replaced daily) and Pheromone (P, same dish kept until pupation). The individuals of the C level were moved to a new clean dish daily at the same time to avoid accumulation of pheromones. The larvae of the pheromone treatments remained in the same dish until pupation to allow accumulation of track compounds—possible pheromones. In both, water was changed, and frozen *Ephestia* eggs ad libitum were replenished each day to minimize cannibalism. Within each dish regime level, three levels of larval density were used (1, 4, 8 larvae per 15 cm Petri dish) forming altogether six combinations: 1 larva living alone (C1, P1), 4 larvae living in a group (C4, P4), and 8 larvae living in a group (C8, P8). We assigned an ID for each Petri dish.

The developmental times of the fourth instar and of the pupa were measured in 12 h intervals and recorded for each individual. The experiment was performed within two months, resulting in 354 larvae measured (Table S1). Within one day after each adult emerged from pupa, the body mass was measured to the precision 0.1 mg. A total of 324 adult individuals from all treatments were examined (Table S2).

The measured variables were analyzed using linear mixed-effects models in Statistica 13 (Tibco 2020) (Tibco, San Ramon, CA, USA). Dish regime (Clean vs. Pheromone) and larval density (1, 4, 8 larvae per dish) and their interaction were included as fixed effects. Petri dish identity was included as a random intercept to account for non-independence of individuals reared within the same dish. Significance of fixed effects was assessed using Type II tests with appropriate denominator degrees of freedom. Post hoc pairwise comparisons of all six combinations of factors were conducted on estimated marginal means with Tukey adjustment for multiple testing. A standard significant difference value of *p* < 0.05 was considered.

## 3. Results

### 3.1. Developmental Time

**Figure 1:**
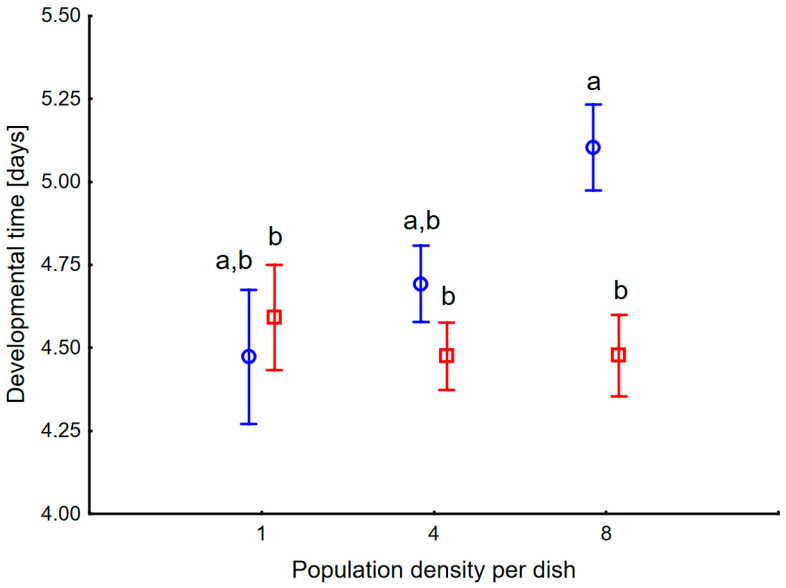
Developmental time of 4th instar larvae of *Harmonia axyridis* maintained in various numbers per 15 cm Petri dish and in Clean level (blue circles), and Pheromone level (red squares). Vertical bars denote 0.95 confidence intervals. Letters indicate comparisons of means using Tukey HSD test among all six combinations.

The fourth instar developmental time differed among factor levels, with significant effects of dish regime, density, and their interaction (Figure 1). Larval developmental time was shorter in P than in the C level (F_1_ = 17.4, *p* = 0.00007). High density of individuals resulted in longer larval development (F_2_ = 4.2, *p* = 0.017), mainly in the C level, as shown by strong interaction (F_2_ = 7.5, *p* = 0.0009). The random effect of the dish was also significant (F_135_ = 2.0, *p* = 0.000004). Developmental time in treatment C8 was longer than in P1, P4, and P8 (*p* = 0.02, 0.04, 0.003). Such a pattern suggests that a high number of physical encounters between larvae in a clean environment prolongs their development, decreasing the risk of predation in the pupal stage. But a high amount of pheromones from larval tracks reverses the effect, keeping the development as fast as in solitary larvae.

Pupal developmental time showed no significant effects of dish regime, density, or their interaction (P vs. C: F_1_ = 0.41, *p* = 0.71, density F_2_ = 0.9, *p* = 0.39, interaction F_2_ = 1.40, *p* = 0.23), suggesting that neither group density nor pheromone action affected development time from pupa to adult. In all cases, the average time was 5.5 days.

### 3.2. Adult Body Mass

**Figure 2:**
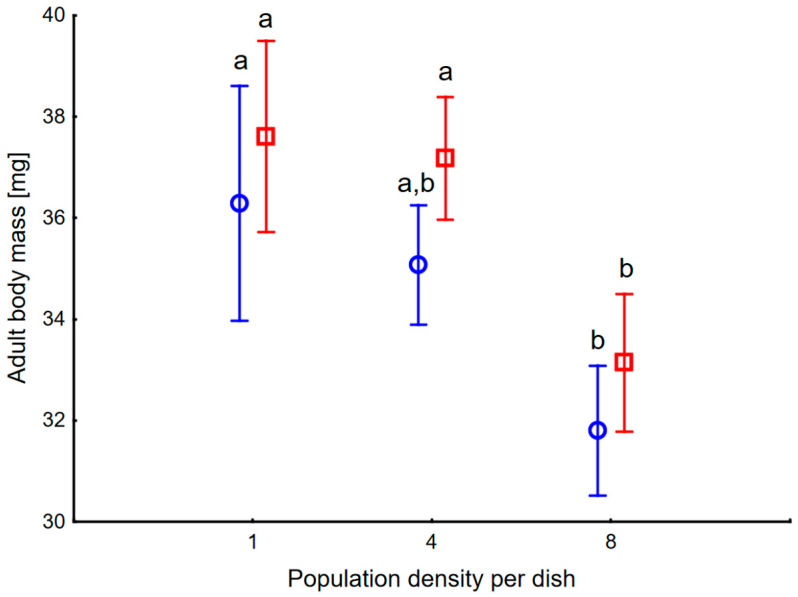
Body mass of one-day-old adults of *Harmonia axyridis* maintained as larvae in various numbers per 15 cm Petri dish and in Clean level (blue circles), and Pheromone level (red squares). Vertical bars denote 0.95 confidence intervals. Letters indicate comparisons of means using Tukey HSD test among all six combinations.

Adult body mass showed significant main effects of dish regime and density, while the interaction was not significant (Figure 2). Body mass was higher in P than in C level (F_1_ = 7.2, *p* = 0.009). High density of larvae resulted in smaller adults (F_2_ = 15.5, *p* < 0.00001), similarly in C and P levels (interaction F_2_ = 0.16, *p* = 0.85). The random effect of the dish was also significant (F_119_ = 1.5, *p* = 0.007). Body mass in combination C8 was lower than in C1 (*p* = 0.04), in P8 lower than in P1 and P4 (*p* = 0.01, 0.03). Such a pattern suggests that a high number of physical encounters between larvae decreases the body mass of resulting adults, regardless of pheromone amount, probably due to lower food intake following stress.

## 4. Discussion

### 4.1. Developmental Time

When population density increased to the highest level, while the amount of putative pheromones was kept low (combination C8), developmental time of the last instar larvae—the most voracious and cannibalistic—significantly increased. Unexpected frequent encounters with conspecifics seemed to delay metamorphosis to avoid becoming a defenseless pupal stage in the presence of many predators. Similar results were found in two Russian populations of *H. axyridis* [22], where an increase in population density (1, 5, and 10 per dish) led to an increase in development time. Other factors had a stronger effect than population density, with long photoperiod (18 h) prolonging development, and diet consisting of aphid *Myzus persicae* accelerated development in comparison to *Ephestia* eggs diet. In other study, the presence of larval tracks of *Coccinella septempunctata* and *Coccinella transversalis* significantly reduced their predation potential and prolonged immature developmental duration [23]. The authors thus propose the compounds to be called feeding deterrent pheromones. We did not observe such effects in our *H. axyridis* population, rather the opposite, which can be attributed to (1) observation of the fourth instar only, (2) the aggressive nature of the invasive populations of *H. axyridis*.

A possible explanation for the differences in development times related to density and pheromones could be that high levels of stress from physical interactions and the presence of other individuals (competitors) may force individuals to compete for available resources (food, space, shelter). In this situation, individuals may prioritize short-term survival over development, taking more time to reach the pupal stage. This explanation was suggested for *Locusta migratoria* (Orthoptera: Acrididae) and *Aedes aegypti* (Diptera: Culicidae), where some of the effects of increased population density could produce immune responses and signs of nutritional deficiencies that affect developmental time [24,25].

The pupal development lasted 5.5 days in all treatments, which corresponds to the values found in the literature, where the time spent in the pupal stage was 5.3 days at 24 °C [26]. There was no delay either due to high density of larvae or pheromones deposited—pupae developed as fast as possible. They do not perceive the two types of information due to their limited sensory capacity, and they do not remember the situation from their previous life.

### 4.2. Adult Body Mass

Body mass of newly hatched adults, which is mostly dependent on mass gain during the last larval instar, also decreased only at the highest level of larval population density (combinations C8, P8). Similar results were found in the two Russian populations [22], where an increase in population density led to a significant decrease in body mass, but only when fed with aphids. Density-dependent factors, such as cannibalism and food availability, negatively affected larval development in both native and invasive Russian *H. axyridis* populations, leading to smaller adults. Because food supply was not limited in our experiments, and it consisted of *Ephestia* eggs, we consider the stress of frequent encountering of the other larvae responsible for the lowering of food ingestion or utilization.

Some authors also propose that competition for space indirectly affects the ability to feed and move efficiently. This trend was observed in the flies *Musca domestica, Drosophila melanogaster*, the tropical butterfly *Bicyclus anynana*, and the moth *Lymantria dispar* [27,28,29,30,31]. In all the cases studied, the population density was manipulated, finding that an increase in population density directly affects stress levels, producing a 10–45% decrease in the body mass of individuals, as well as other factors such as fertility and the effectiveness of sexual reproduction.

Ladybird females are always heavier than males [32], which was not distinguished in our samples. However, we found much higher average fresh mass than that reported for *H. axyridis* 28.3 ± 2.8 mg at long photoperiod females fed with *M. persicae* [33]. This difference suggests that food stress was not the direct driver of differences among our conditions.

However, the effects of density and pheromones were not positively combined. Perception of larval tracks (P level) increased the adult body mass. A possible mechanism of such increase could be that larval feeding and utilizing the food for growth was improved compared to clean environment (C level), contradicting results reported in two *Coccinella* species [23]. Because each larva produces and then feels its own pheromones, it seems to be calmed from a possible stress in their presence. Known, already visited vegetation can be safer than an unknown one.

### 4.3. General Discussion

The trends found in this study caused by population density and deposited pheromones in both development time and body mass in *H. axyridis* warrants further investigation. Future studies should conduct a detailed analysis by increasing population density even further and observing changes in developmental time, body mass, and survival rate to determine a possible self-regulation mechanism of the species field populations at high densities. However, the effect of high larval density can be diminished in nature, where physical contact or encounters between individuals may cause them to take opposite paths and directions to avoid interaction and keep distance from each other. Random effects of the dishes where larvae were kept together were large; therefore, we interpret pairwise contrasts cautiously, focusing on the interaction pattern. The effect was really random; no systematic factors responsible for the variations were found. However, we recommend keeping possible variability of conditions at a minimum by making similar experiments during a short time with a genetically homogeneous strain.

### 4.4. Limitations of the Study

Considering the scenario where an individual enters the pupal stage while surrounded by individuals in the larval stage, this individual would be defenseless and immobile and could be cannibalized. Both the stress caused by the encounters leading to inefficient food intake and utilization and the endeavor to avoid the defenseless stage are logical interpretations of our results. But we cannot define the mechanisms of observed buffering density-induced stress, because no feeding, metabolic, or hormone-related measurements were conducted. We continue with the experimentation, including GC-MS analyses of the tracks, but in the present study, no chemical identification or quantification was performed, and alternative sources of chemical cues cannot be excluded. The used 12 h observation interval is still not common in related studies, although more usual once-daily observation does not allow the finding of small differences between conditions. Besides the precision of data, there are other potential interval-censoring effects such as circadian rhythm in pupation [34].

High density may begin to self-regulate populations due to negative effects on growth and survival, reducing the risk of overpopulation. The presence of pheromones without physical contact with larvae could induce behaviors or physiological responses consistent with a buffering of density-related delay (without direct evidence of acceleration mechanisms), due to the activation of species regulation mechanisms.

## 5. Conclusions

Larvae of ladybirds seem to react both to their density and to the presence of larval tracks. High larval density had negative effects on developmental rate and subsequent adult body size. However, the presence of the tracks stabilized the high developmental rate and increased body size. Thus, the compounds present in the tracks, previously known as oviposition-deterring pheromone, can be also viewed as having a potential buffering effect that may mitigate density-induced developmental delay.

## Data Availability

The original contributions presented in this study are included in the Supplementary Materials. Further inquiries can be directed to the corresponding author.

## Supplementary Materials

The following supporting information can be downloaded at: https://www.mdpi.com/article/10.3390/insects17030317/s1, Table S1: Fernandez_developmental_time; Developmental time in days with dish regime (C: clean, P: pheromone), number of larvae per dish and identity of dish. Table S2: Fernandez_body_mass. Adult body mass in mg with dish regime (C: clean, P: pheromone), number of larvae per dish and identity of dish.
