# Supplementary material for: Effect of a Marking Pheromone and Population Density on Ladybird Larval Development and Adult Body Mass
Source: Insects. 2026 Mar 16;17(3):317. doi: 10.3390/insects17030317 (PMC13026648; doi:10.3390/insects17030317)
Supplement: Supplementary file 1 [file insects-17-00317-s001.zip › Table S1. Fernandez_developmental_time.pdf]

Pheromone	Density	Dish_id	Larval developmental time [d]
C	1	C10	4,5
C	1	C11	4,5
C	1	C12	4,5
C	1	C13	4,5
C	1	C14	4,4
C	1	C15	4,4
C	1	C16	4,4
C	1	C17	4,4
C	1	C18	4,4
C	1	C19	5,0
C	1	C2	4,0
C	1	C20	5,0
C	1	C21	4,8
C	1	C22	4,8
C	1	C23	4,8
C	1	C24	4,8
C	1	C25	5,5
C	1	C3	3,9
C	1	C4	3,9
C	1	C5	3,9
C	1	C6	3,9
C	1	C7	3,9
C	1	C8	4,5
C	1	C9	4,5
C	4	C1	3,0
C	4	C1	4,0
C	4	C1	4,0
C	4	C1	4,5
C	4	C34	3,5
C	4	C35	4,4
C	4	C38	4,0
C	4	C38	4,5
C	4	C38	4,4
C	4	C38	5,0
C	4	C39	4,0
C	4	C39	4,5
C	4	C39	5,0
C	4	C39	5,0

C	4	C40	4,0
C	4	C40	4,0
C	4	C41	4,0
C	4	C41	4,5
C	4	C41	4,8
C	4	C42	4,0
C	4	C42	5,0
C	4	C42	5,0
C	4	C43	4,0
C	4	C43	3,9
C	4	C43	4,8
C	4	C44	4,0
C	4	C44	4,5
C	4	C44	4,5
C	4	C45	4,5
C	4	C45	4,8
C	4	C45	4,8
C	4	C45	4,8
C	4	C46	4,5
C	4	C46	5,0
C	4	C47	4,5
C	4	C47	4,8
C	4	C48	5,0
C	4	C48	4,8
C	4	C48	4,8
C	4	C48	4,8
C	4	C49	5,0
C	4	C49	5,0
C	4	C49	5,0
C	4	C50	5,0
C	4	C50	4,8
C	4	C51	5,0
C	4	C51	4,8
C	4	C51	4,8
C	4	C51	4,8
C	4	C52	5,0
C	4	C53	5,0
C	4	C53	4,8
C	4	C54	5,0
C	4	C54	5,5
C	4	C55	5,0
C	4	C55	4,8
C	4	C55	4,8
C	4	C55	4,8
C	4	C56	4,8
C	4	C56	5,5
C	4	C57	4,8
C	4	C57	4,8

C	4	C57	4,8
C	4	C58	4,8
C	4	C58	5,5
C	4	C58	5,3
C	4	C59	4,8
C	4	C60	4,8
C	4	C61	4,8
C	4	C61	4,8
C	4	C62	4,8
C	4	C62	5,5
C	4	C63	4,8
C	4	C63	5,5
C	8	C26	3,5
C	8	C26	4,0
C	8	C26	4,5
C	8	C26	4,5
C	8	C26	5,0
C	8	C26	5,0
C	8	C26	5,0
C	8	C26	5,5
C	8	C27	4,0
C	8	C27	4,5
C	8	C27	4,5
C	8	C27	5,0
C	8	C27	5,0
C	8	C27	5,0
C	8	C28	4,0
C	8	C28	5,0
C	8	C28	5,0
C	8	C28	5,0
C	8	C28	5,5
C	8	C28	6,0
C	8	C29	4,0
C	8	C29	4,5
C	8	C29	4,5
C	8	C29	4,5
C	8	C29	4,5
C	8	C30	4,5
C	8	C30	4,5
C	8	C30	4,5
C	8	C30	5,0
C	8	C30	5,5
C	8	C31	4,5
C	8	C31	5,5
C	8	C31	5,5
C	8	C31	5,5
C	8	C31	5,5
C	8	C31	6,0

C	8	C32	4,5
C	8	C32	5,5
C	8	C32	6,0
C	8	C32	6,0
C	8	C33	4,5
C	8	C33	5,5
C	8	C33	5,5
C	8	C33	5,5
C	8	C33	5,5
C	8	C33	5,5
C	8	C34	5,5
C	8	C34	5,5
C	8	C34	6,0
C	8	C35	5,5
C	8	C35	6,0
C	8	C35	6,0
C	8	C36	5,5
C	8	C36	5,5
C	8	C36	5,5
C	8	C36	5,5
C	8	C37	5,5
C	8	C45	6,0
P	1	P27	4,0
P	1	P28	4,0
P	1	P29	4,0
P	1	P30	4,0
P	1	P31	4,0
P	1	P32	3,9
P	1	P33	4,5
P	1	P34	4,5
P	1	P35	4,5
P	1	P36	4,5
P	1	P37	4,5
P	1	P39	4,4
P	1	P40	4,4
P	1	P41	4,4
P	1	P42	4,4
P	1	P43	4,4
P	1	P44	4,4
P	1	P45	4,4
P	1	P46	4,4
P	1	P47	4,4
P	1	P48	4,4
P	1	P49	4,4
P	1	P50	5,0
P	1	P51	5,0
P	1	P52	4,8
P	1	P53	4,8

P	1	P54	4,8
P	1	P55	4,8
P	1	P56	4,8
P	1	P57	4,8
P	1	P58	4,8
P	1	P59	4,8
P	1	P60	4,8
P	1	P61	4,8
P	1	P62	4,8
P	1	P63	5,5
P	1	P64	5,5
P	1	P65	5,3
P	1	P66	5,3
P	4	P10	4,0
P	4	P10	4,0
P	4	P10	3,9
P	4	P11	4,0
P	4	P11	4,5
P	4	P11	4,5
P	4	P12	4,0
P	4	P12	4,5
P	4	P12	4,5
P	4	P12	4,5
P	4	P13	4,0
P	4	P13	4,0
P	4	P13	4,5
P	4	P14	4,0
P	4	P14	4,0
P	4	P15	4,0
P	4	P15	4,0
P	4	P15	4,4
P	4	P15	4,8
P	4	P16	4,0
P	4	P16	4,5
P	4	P16	4,5
P	4	P16	4,5
P	4	P17	4,5
P	4	P17	4,4
P	4	P17	4,4
P	4	P17	4,4
P	4	P18	4,0
P	4	P18	4,4
P	4	P18	6,0
P	4	P19	4,0
P	4	P19	4,0
P	4	P19	4,0
P	4	P20	4,0
P	4	P20	4,5

P	4	P20	4,4
P	4	P21	4,0
P	4	P21	4,0
P	4	P21	4,5
P	4	P21	4,4
P	4	P22	4,0
P	4	P22	4,0
P	4	P22	4,0
P	4	P22	4,4
P	4	P23	4,0
P	4	P23	4,4
P	4	P23	4,4
P	4	P23	4,4
P	4	P24	4,0
P	4	P24	4,5
P	4	P24	4,4
P	4	P25	4,0
P	4	P25	4,4
P	4	P25	4,4
P	4	P25	4,4
P	4	P26	4,0
P	4	P26	4,4
P	4	P26	4,4
P	4	P26	4,4
P	4	P69	4,4
P	4	P69	4,4
P	4	P69	4,4
P	4	P69	5,0
P	4	P70	4,4
P	4	P70	5,0
P	4	P70	5,0
P	4	P70	5,0
P	4	P71	4,4
P	4	P71	4,4
P	4	P71	5,5
P	4	P71	5,5
P	4	P72	4,4
P	4	P72	4,4
P	4	P72	5,0
P	4	P72	5,0
P	4	P73	4,4
P	4	P73	4,4
P	4	P73	4,4
P	4	P74	4,4
P	4	P74	5,0
P	4	P74	5,0
P	4	P75	4,4
P	4	P75	4,8

P	4	P75	4,8
P	4	P75	6,0
P	4	P76	4,4
P	4	P76	5,3
P	4	P76	5,3
P	4	P76	5,3
P	4	P77	4,4
P	4	P77	5,3
P	4	P77	5,3
P	4	P78	4,8
P	4	P78	5,3
P	8	P1	3,5
P	8	P1	3,5
P	8	P1	4,0
P	8	P1	4,0
P	8	P1	4,0
P	8	P1	4,0
P	8	P1	4,5
P	8	P1	4,5
P	8	P2	3,5
P	8	P2	4,0
P	8	P2	4,5
P	8	P2	4,5
P	8	P2	4,5
P	8	P3	3,5
P	8	P3	4,0
P	8	P3	4,0
P	8	P4	3,5
P	8	P4	4,5
P	8	P4	4,5
P	8	P4	4,5
P	8	P4	4,5
P	8	P4	4,5
P	8	P5	3,5
P	8	P5	3,5
P	8	P5	4,0
P	8	P5	4,5
P	8	P5	4,5
P	8	P5	5,0
P	8	P5	5,0
P	8	P6	3,5
P	8	P6	4,5
P	8	P6	5,0
P	8	P6	5,0
P	8	P6	5,0
P	8	P6	6,0
P	8	P6	6,0
P	8	P67	4,5

P	8	P67	4,5
P	8	P67	4,5
P	8	P67	4,5
P	8	P67	4,5
P	8	P67	4,5
P	8	P68	4,5
P	8	P68	4,5
P	8	P68	4,5
P	8	P7	4,5
P	8	P7	4,5
P	8	P7	4,5
P	8	P7	4,5
P	8	P7	5,0
P	8	P7	5,0
P	8	P8	4,0
P	8	P8	4,0
P	8	P8	4,5
P	8	P8	4,5
P	8	P8	5,0
P	8	P8	5,0
P	8	P8	5,0
P	8	P8	5,0
P	8	P9	4,0
P	8	P9	5,0
P	8	P9	5,0
P	8	P9	5,0
P	8	P9	5,5
P	8	P9	5,5
