# Supplementary material for: Effect of a Marking Pheromone and Population Density on Ladybird Larval Development and Adult Body Mass
Source: Insects. 2026 Mar 16;17(3):317. doi: 10.3390/insects17030317 (PMC13026648; doi:10.3390/insects17030317)
Supplement: Supplementary file 1 [file insects-17-00317-s001.zip › Table S2. Fernandez_body_mass.pdf]

Pheromone	Density	Dish_id	Adult body mass [mg]
C	1	C16	36,2
C	1	C17	34,2
C	1	C18	32,8
C	1	C19	32,7
C	1	C20	42,8
C	1	C21	36,5
C	1	C34	41,8
C	1	C35	34,1
C	1	C36	30,7
C	1	C37	42,6
C	1	C38	44,4
C	1	C39	39,1
C	1	C40	39,3
C	1	C41	31,5
C	1	C42	32,9
C	1	C43	40,5
C	1	C44	30
C	1	C45	35,9
C	1	C46	39,1
C	1	C47	28,8
C	4	C1	34,8
C	4	C1	29,2
C	4	C1	32,9
C	4	C10	31,1
C	4	C10	28,7
C	4	C10	30,6
C	4	C11	35,7
C	4	C11	32,4
C	4	C11	34
C	4	C11	27
C	4	C12	35,2
C	4	C13	36,8
C	4	C13	27,5
C	4	C14	36
C	4	C14	40,8
C	4	C15	52,3
C	4	C15	23,3
C	4	C2	35,2
C	4	C2	36,2
C	4	C22	42,9
C	4	C22	24,3
C	4	C22	24,7
C	4	C23	39,7

C	4	C23	34,1
C	4	C24	32
C	4	C24	33,8
C	4	C24	38
C	4	C24	32,8
C	4	C25	32,5
C	4	C25	35,3
C	4	C25	32
C	4	C26	36,1
C	4	C26	33,6
C	4	C26	36,7
C	4	C27	31,8
C	4	C27	38,8
C	4	C27	36,1
C	4	C28	42
C	4	C28	41,8
C	4	C29	34
C	4	C29	32
C	4	C29	40,3
C	4	C29	34,2
C	4	C3	31,5
C	4	C3	36,1
C	4	C3	34,3
C	4	C3	35
C	4	C30	42,7
C	4	C30	32,7
C	4	C30	38,2
C	4	C30	37,1
C	4	C31	39,7
C	4	C31	44,2
C	4	C31	38,2
C	4	C31	37
C	4	C32	32
C	4	C32	33,4
C	4	C32	43,2
C	4	C33	42,4
C	4	C33	36,4
C	4	C4	32,5
C	4	C4	27
C	4	C5	31,3
C	4	C5	38
C	4	C5	35,7
C	4	C6	30
C	4	C6	33,9
C	4	C6	40
C	4	C7	46,5
C	4	C7	28,5
C	4	C7	35
C	4	C7	48,3
C	4	C8	43,9

C	4	C8	42,5
C	4	C9	21,6
C	4	C9	40,1
C	4	C9	26,1
C	4	C9	21,5
C	8	C48	39,1
C	8	C48	38
C	8	C48	37
C	8	C48	33,3
C	8	C48	34,8
C	8	C48	40,2
C	8	C49	39,3
C	8	C49	44,8
C	8	C49	35,5
C	8	C49	33
C	8	C49	40,8
C	8	C50	31,2
C	8	C50	33,6
C	8	C50	33,5
C	8	C50	31,2
C	8	C50	34,7
C	8	C51	27,4
C	8	C51	26,6
C	8	C51	29,5
C	8	C51	34,1
C	8	C51	26
C	8	C51	30,6
C	8	C51	35,7
C	8	C52	33,9
C	8	C52	24,2
C	8	C52	30,4
C	8	C52	32
C	8	C52	28,9
C	8	C52	28,1
C	8	C53	26,8
C	8	C53	33,3
C	8	C53	26,5
C	8	C53	30,7
C	8	C54	36,5
C	8	C54	25,5
C	8	C54	34,8
C	8	C54	30,9
C	8	C54	27,6
C	8	C54	33,5
C	8	C54	31,7
C	8	C55	25,1
C	8	C55	28
C	8	C55	27,2
C	8	C55	33,6
C	8	C55	32,4

C	8	C55	37,7
C	8	C56	29,3
C	8	C56	29,6
C	8	C56	24,6
C	8	C56	28,8
C	8	C56	30,1
C	8	C57	32,2
C	8	C57	26,5
C	8	C57	27,2
C	8	C57	33,8
C	8	C58	41,7
C	8	C58	39,3
C	8	C58	30,2
C	8	C58	26,5
C	8	C58	28,4
C	8	C58	40,7
C	8	C59	23
C	8	C59	28,5
C	8	C59	31,9
C	8	C59	25,5
P	1	P1	29,4
P	1	P10	31,5
P	1	P11	33,4
P	1	P12	44,2
P	1	P13	40,8
P	1	P14	38,7
P	1	P16	39,3
P	1	P17	47,7
P	1	P18	50,5
P	1	P2	38,4
P	1	P3	42,5
P	1	P37	30,2
P	1	P38	37,7
P	1	P39	28
P	1	P40	32,3
P	1	P41	39,3
P	1	P42	33
P	1	P43	45,2
P	1	P44	35,1
P	1	P45	35,2
P	1	P46	29,5
P	1	P47	37,1
P	1	P48	36,6
P	1	P49	38,4
P	1	P50	36,6
P	1	P51	40,3
P	1	P52	39,8
P	1	P53	46,8
P	1	P54	32
P	1	P9	38,8

P	4	P15	43,6
P	4	P15	34,9
P	4	P19	36,2
P	4	P19	38,1
P	4	P19	48,5
P	4	P19	50,9
P	4	P20	38,2
P	4	P20	43,3
P	4	P20	49
P	4	P21	36,3
P	4	P21	41,4
P	4	P22	33,7
P	4	P22	34,7
P	4	P22	36
P	4	P22	34,9
P	4	P23	33,9
P	4	P23	35,5
P	4	P23	45,2
P	4	P24	32
P	4	P24	25,9
P	4	P24	40,8
P	4	P25	32,7
P	4	P25	35,5
P	4	P25	32,4
P	4	P25	33,6
P	4	P26	39,7
P	4	P26	31,3
P	4	P27	36,3
P	4	P27	31,2
P	4	P27	29,4
P	4	P27	32,3
P	4	P28	32,1
P	4	P28	37,7
P	4	P28	41,3
P	4	P29	40,4
P	4	P29	37,6
P	4	P29	34,1
P	4	P30	32,1
P	4	P30	34,8
P	4	P30	42,2
P	4	P31	40,1
P	4	P31	43
P	4	P32	39,6
P	4	P32	37,1
P	4	P32	29,8
P	4	P32	39,7
P	4	P33	36,9
P	4	P33	26,4
P	4	P33	38
P	4	P34	31,2

P	4	P34	45,6
P	4	P34	41,5
P	4	P34	36
P	4	P35	34,8
P	4	P35	38,1
P	4	P35	35,2
P	4	P36	32,9
P	4	P36	32,6
P	4	P4	32,7
P	4	P4	41,3
P	4	P4	44
P	4	P5	47,2
P	4	P5	36,8
P	4	P5	36,6
P	4	P6	39,8
P	4	P6	44,8
P	4	P7	36,8
P	4	P7	36,6
P	4	P7	42,3
P	4	P7	33
P	4	P8	40
P	4	P8	33
P	4	P8	31
P	8	P55	35,3
P	8	P55	35,9
P	8	P55	30,7
P	8	P55	38,4
P	8	P55	30,4
P	8	P55	39,2
P	8	P56	29
P	8	P56	31,7
P	8	P56	27,2
P	8	P56	40,2
P	8	P56	28,4
P	8	P56	32,8
P	8	P56	25,8
P	8	P57	26,8
P	8	P57	37
P	8	P57	29,6
P	8	P57	33,2
P	8	P57	29,6
P	8	P57	31,4
P	8	P58	36
P	8	P58	31,4
P	8	P58	33,1
P	8	P58	37
P	8	P58	31,1
P	8	P59	40,7
P	8	P59	37,9
P	8	P59	31,8

P	8	P59	30,1
P	8	P59	36,6
P	8	P60	30,7
P	8	P60	32,3
P	8	P60	30,6
P	8	P60	28,7
P	8	P61	32,4
P	8	P61	27,6
P	8	P61	37,9
P	8	P61	33
P	8	P61	33,7
P	8	P61	42
P	8	P62	26,3
P	8	P63	29,5
P	8	P63	27,5
P	8	P63	29
P	8	P63	32,8
P	8	P64	40,7
P	8	P64	34,1
P	8	P64	33,7
P	8	P64	30,7
P	8	P64	30,7
P	8	P64	41,3
P	8	P64	42,9
P	8	P65	25,6
P	8	P65	32,6
P	8	P65	40
P	8	P65	35,5
P	8	P66	44,7
P	8	P66	34,4
P	8	P66	23
